# Marketing data: Has the rise of impact factor led to the fall of objective language in the scientific article?

**DOI:** 10.1186/1465-9921-10-35

**Published:** 2009-05-11

**Authors:** Véronique J Fraser, James G Martin

**Affiliations:** 1 Meakins-Christie Laboratories, McGill University, Montreal, Québec, Canada

## Abstract

The language of science should be objective and detached and should place data in the appropriate context. The aim of this commentary was to explore the notion that recent trends in the use of language have led to a loss of objectivity in the presentation of scientific data. The relationship between the value-laden vocabulary and impact factor among fundamental biomedical research and clinical journals has been explored. It appears that fundamental research journals of high impact factors have experienced a rise in value-laden terms in the past 25 years.

## Introduction

A recent editorial addressing the care which must be taken in the reporting of clinical results concluded: "The numbers and not their interpretation, must speak for themselves" [1]. This statement succinctly expresses that which is often taken for granted in scientific research articles; a commitment to the standard of objectivity. Insofar as the scientific article is the principal forum for the dissemination of new knowledge it must reflect a detached and objective set of arguments supported by data and leading to reasonable conclusions [2]. The role of the author is to record, evaluate and situate new evidence within the context of existing scientific literature. It is generally agreed that subjective interpretation of results ought to be minimal and tempered with discretion. Yet, we have noted adjectives imposing subjective value on an otherwise neutral knowledge claim appearing with increasing frequency in the scientific literature. Readers of scientific articles currently encounter frequent claims of "crucial", "critical" or "unique" events as well as "important" or "original" discoveries. The hypothesis that the language of science has changed to include words which might potentially bias the reader in his/her interpretation of the research article has prompted us to conduct an investigation into what appeared to be a shift in the use of language in scientific articles.

We evaluated this hypothesis by examining twelve established biomedical and fundamental clinical and clinical research journals over a twenty year time period for adjectives which modified an otherwise neutral knowledge claim. Our findings indicate that there is an increase in value-laden language in the scientific article from 1985 to 2005. Both high and low impact fundamental research journals exhibit an increase in biased word choice over time, this trend being most marked in high-impact biomedical journals devoted to fundamental research. Comparatively, clinical journals showed a low incidence of biased words and this characteristic has remained consistent over the time period under investigation. We suggest that the increase in incidence of biased language may provide a means through which to view broader changes occurring within the scientific community. Publication practice has evolved over the past twenty years as authors face increasing pressure to publish in high impact journals [1]. While a definitive causal link between current publication pressure and biased word choice cannot be established by our data; we believe that an analysis such as ours raises some pertinent questions about publication practice as it exist today.

## Methods: evaluating the use of language

To assess the use of value-laden language we began by formulating a list of adjectives we had noticed appearing with increasing frequency in the scientific literature. The list of words compiled, while by no means exhaustive, reflects a general sampling of adjectives which attribute status or significance to an otherwise neutral claim (Table 1). Words were subsequently divided into two categories and given an arbitrary weighted score based on their inherent impact and ability to induce bias in the reader. For example, we operated on the belief that an enzyme described as 'critical" or 'crucial" may reasonably be assumed to be of greater significance than an "important" enzyme, which is presumably in turn more significant than an enzyme with no descriptive claim at all. As such, words in the former category were allotted a biased word score of three, while those in the latter were assigned a biased word score of one. We subsequently selected twelve journals in the following two categories: Medicine: Research and Experimental and Medicine, General and Internal. The journals were chosen with the following criteria in mind; first, they reflect the informal hierarchy assigned by Impact Factor (I.F.), ranging from the low to high end of the spectrum. Specifically, we used four journals with an I.F. between zero and five, 5 journals with an I.F. between five and twenty and 3 journals with an I.F between twenty-five and forty-five (Table 1). Journals with an I.F. of four or less were classified as Low Impact while those greater than four were classified as High Impact. We were careful that the Low Impact Journals selected were both well regarded and well read publications. Second, we chose journals that represent both fundamental (7 journals) and clinical research (5 journals) with journals classified as high or low Impact represented in each category. We then evaluated the changing use of language over a twenty year time period, selecting three time points; 1985, 1995 and 2005 for comparison. Five original, disparately cited research articles were selected at random from each journal and analyzed using Optical Character Recognition (OCR) software for the adjectives listed in Table 1.

**Table 1:** Journals

New England Journal of Medicine	44.016
Science	30.927
Nature Medicine	28.878
Journal of Clinical Investigation	15.053
Journal of Experimental Medicine	13.965
British Medical Journal	9.752
Canadian Medical Association Journal	7.402
Journal of Immunology	6.387
Journal of Pharmacology	4.098
American Journal of Physiology	3.942
Laboratory Investigations	3.859
European Journal of Clinical Investigation	2.537

As the presence or absence of biased language is context dependent, some subjective evaluation was necessary in the tabulation of biased word count. Once OCR identified an adjective, one of the authors (V.F.), used the following guidelines to determine whether a word could be judged to modify the content of the sentence. Selected words (Table 2) were exempt from tabulation if they failed to modify the knowledge claims posited by the paper. For example, the word "major" would be counted as ascribing bias in the following sentence: "...Complex A plays a major role in calcium signaling" but ignored in the following: "Substrate A targets the major binding site." Further exemptions include "paradigm" when not accompanied by "new", "shift", "change" etc.; the word "vital" was ignored when joined to "capacity", while "central" was omitted if it modified "thesis", "argument" etc. Words that accompanied information cited in other articles, for example "Dawson et. al, demonstrated the novel use of placebo A in case X" were exempt from tabulation in order to ensure consistency. Furthermore, we limited the scope of our investigation to words used in a positive context. The word "important" was ignored if it appeared in a sentence as "not important". Similarly, the word "definitive" was counted only if it was used to make a positive claim about knowledge put forward in the paper. It was ignored in the following sentence "Further research must be undertaken before a definitive claim can be made..." These considerations allowed us to tabulate a final biased word score indicative of the presence of language with the potential to bias or impose value judgment on the reader.

**Table 2:** Words scored

Bias Factor 3	Bias Factor of 1	
Pivotal	Important	New
Crucial	Innovative	Novel
Critical	Central	First (demonstration)
Vital	Definitive	Direct mechanistic
Unique	Necessary	Powerful
Essential	Advance	
Paradigm (Change)	Major	
Key	Noteworthy	

## Results

**Figure 1:**
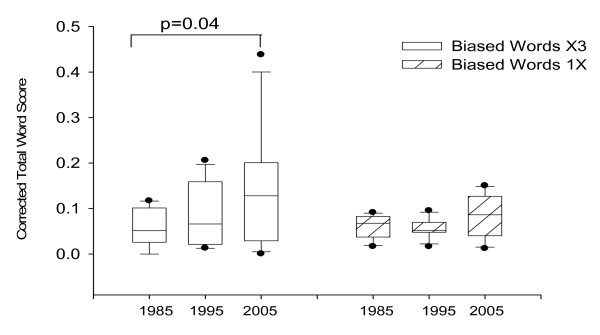
**Increase in biased words in the last twenty years**. The data are normalized by dividing the total word score obtained for an article for biased words by the total number of words contained in the same article The median, 25th, 75th, percentiles are shown. Statistical significance was assessed by Student T-test and corrected for multiple comparisons.

**Figure 2:**
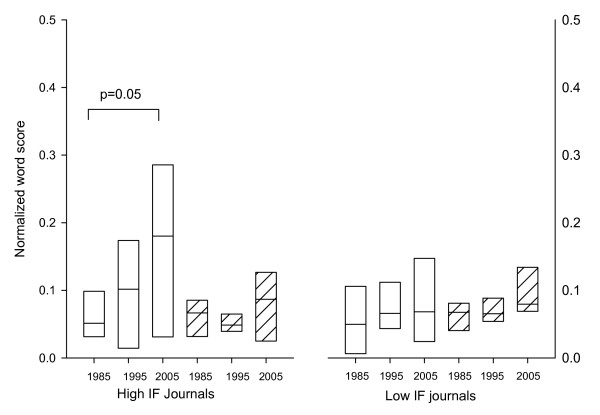
**Content of biased words in low versus high impact journals**. The data are normalized by dividing the total word score obtained for an article by the total number of words contained in that same article. The median, 25th, 75th, percentiles are shown. The data show that there was a significant increase in the use of highly biased words over the past 20 years in high impact journals but not in low impact journals. Statistical significance was assessed by Student T-test and corrected for multiple comparisons.

A number of clear trends emerged from the analysis. Firstly, for all journals there was an increase in the word score (points accumulated for biased words/total number of words in article) from 1985 through to 2005 (Figure 1.) The trend was more obvious for words that we judged to merit a weighting of 3. The trend was weaker for words with a weighting of 1. When the journals were separated into high versus low impact factor journals there was a striking difference (figure 2); high impact journals were more subject to the change in language.

**Figure 3:**
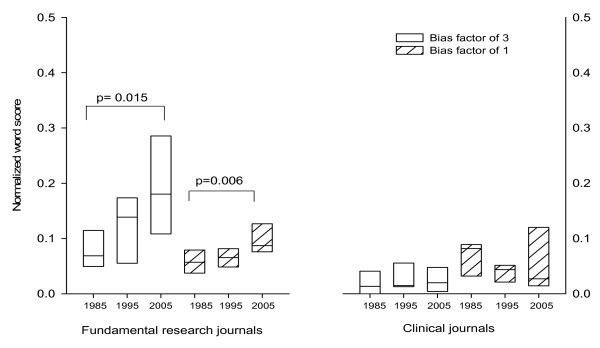
**Content of biased words in fundamental versus clinical research journals**. The data are normalized by dividing the total word score obtained for an article by the total number of words contained in that same article. The median, 25th, 75th, percentiles are shown. The data show that there was a significant increase in the use of biased words over the past 20 years in the fundamental science journals but not in clinical science journals Statistical significance was assessed by Student T-test and corrected for multiple comparisons.

The use of biased language in clinical journals was infrequent, with no increase in the use of value-laden words over the twenty year interval (figure 3). When the fundamental journals and clinical journals were partitioned into high and low impact the increase in word scores for the group overall was clearly attributable to the vocabulary employed in the fundamental journals of high impact.

## Discussion

The increasing incidence of adjectives expressing subjective judgments undermines what has traditionally been accepted as the objective nature of the scientific paper. Our argument therefore assumes that objectivity is an integral and necessary component in the quest for scientific progress. Most would tacitly acknowledge that objectivity occupies a unique position within scientific disciplines. In his paper: *The Scope and Limits of Scientific Objectivity *Joseph F. Hannah states: "It is generally agreed that one of the distinguishing virtues of science is its objectivity. The scope of science is the *objective world *and the limits of science are determined by the limits of the *objective methods *of formal and empirical research" [3]. Insofar as the scientific paper is the primary vehicle for new and private scientific findings to enter into the realm of public discourse, it should also demonstrate a commitment to the principles and standards of objectivity. We would argue that the paper may take a subjective stance insofar as it argues for the relevance of the observations it posits as well as to the implications the observation will have on the established body of knowledge, but these contextual arguments should be minimal and tempered with discretion. The strength and import of observations and conclusions should be evident in and of themselves with minimal positioning on the part of the authors.

The demonstrable increase in the use of adjectives with the potential to bias the reader may indicate that the interpretation of results has come to replace what has traditionally been a more objective stance. This shift towards the somewhat hyperbolic interpretation of data from the more conservative representation of data, raises important questions about the evolution of the scientific article and must be examined in conjunction with changing attitudes within the scientific community regarding the writing and submission of articles, the mounting impact of the impact factor and the pressures currently facing authors seeking publication.

### The Rising Impact of the Impact Factor

Changing attitudes towards scientific publication must be examined in tandem with the changing role of the impact factor in assessing the merits of a body of work and the "impact" this has had on the scientific community. Briefly, the impact factor of a journal reflects the number of citations appearing in indexed publications in a given year to articles published in a given journal in the previous two years, divided by the number of citable papers published within these two years. However, the original purpose of the database developed by the Institute for Scientific Information and used for citation analysis has been somewhat forgotten and the impact factor has taken on a life of its own. Several detailed critiques of the impact factor have been published [2], highlighting shortcomings such as the limitations of the impact factor in comparisons of journals involving different research fields. In addition, even within a discipline the impact factor may not measure appropriately the quality of the journal. For example, it is sensitive to whether an area of research is young and developing, and therefore likely to lead to citations that are recent, or more mature.

Although the merit of impact factor remains the subject of intense debate, its current influence on scientific papers and publication is not. Impact Factor has extended its reach to be included in the evaluation of academic and medical institutions as well as in the evaluation of researchers for tenure and promotion and the awarding of grants [1]. The latter often hinges not only on the number of publications and the quality of the research but also the impact factor of the journal. In 2002 a Nature News feature noted: "...the implicit use of journal impact factors by committees determining promotions and appointments is endemic" [4]. Similarly, a 1997 British Medical Journal article claimed: "The increasing awareness of journal impact factors and the possibility of their use in evaluation are already changing scientists' publication behaviors towards publishing in journals of maximum impact" [5]. Moreover, the pressure currently facing researchers to publish in high impact journals is in stark contrast to publication behavior as recently as 25 years ago. An investigation undertaken in 1984 into which factors influenced scientists' selection of journals for publication concluded: "... that journals were primarily selected on the basis of the audiences they reach, rather than the rewards they confer, and the reward seeking model of selection behavior found little or no support" [6]. It is interesting to note that the twenty years in which our data demonstrates an increase in biased language corresponds to a time period wherein scientific authors began to change their behaviors with regards to publication. We suggest that the emergence of a new trend in which a reward-seeking-model (high impact factor) begins to supercede target audience as the primary motivation in the selection of journals should not pass unnoticed.

### Scientists' response to the barriers to publication

The status of scientific journals is measured by the impact factor and journal editors have adopted strategies to enhance the impact factor, e.g. by publishing review articles which tend to be cited frequently. Editorial evaluation of articles and their potential acceptance or rejection based on priority is based on interest to the readership, and not necessarily the quality of the science. Rejection of an article based on being low priority for the journal is often not reflected in the reviews provided to the authors. A judgment of low priority is a subjective opinion and as such is not an issue for debate. How "hot" a topic is, is of *critical *importance to its chances of publication. This trend, when examined in conjunction with the increased use of biased words, raises some fundamental questions. Does a reward-seeking-model of publication – as reflected in the current desire to publish in high impact journals – influence the use of language in scientific manuscripts? For instance, is it possible that authors have discovered that an effective strategy to counter the failure of reviewers to be excited about an article is to create bias through the use of language that exaggerates the importance of the findings? Or, is it merely that language exists in a state of flux and any changes in style or vocabulary merely reflect time-related alterations in writing? Finally, perhaps the biased words are not so much biased as emphatic, though necessary, descriptors of the work which is being presented?

At first glance it seems plausible to state that the words under investigation are not reflective of bias, but are rather necessary descriptive terms of what is, in fact, a new and important knowledge claim. A detailed discussion as to whether manuscripts in high impact factor journals are truly more "important" or "novel" than those in low impact journals is beyond the scope of this paper and may be a subject for future investigation. However, we would argue that it is remarkable that the use of biased words has shown an increase over time in both low and high impact journals. That is, it seems unlikely that the ideas posited in scientific articles in 2005 are markedly more valuable or significant than those put forward in 1985. A more plausible explanation is that it is the style, rather than the substance of the articles, which has altered.

It is a truism to state that language is constantly evolving and it seems reasonable to consider the possibility that changes in style and vocabulary may simply reflect time-related alterations in writing. Still, it is interesting that the difference between the language used in fundamental and clinical journals is so marked, with biased words more frequently found in high impact fundamental journals. This prompts the question: why is it that language has only "evolved" in fundamental journals? A hypothesis which suggests itself is that the language used in the interpretation of data in clinical journals has the potential to impact upon clinical practice and is therefore more likely to be tempered than language used in fundamental journals. Be that as it may, the question remains as to why the use of biased language is on the rise in fundamental journals and whether this trend should continue unchallenged. Furthermore, what conclusions may be drawn from grandiloquence and high impact factors? Perhaps it is possible that high rejection rates by editors without the use of peer review increases the pressure for hyperbole so as to clear the first hurdle

## Conclusion

The increased use of biased words provides an interesting locus for a discussion on the changing trends in publication and the increasing pressure felt by authors today. While we hesitate to suggest that the latter is responsible for the former we are confident in the assertion that the use of biased words in a scientific manuscript does not serve a useful purpose. The readership is unlikely to require orientation to ensure that pivotal and central observations pass unrecognized inadvertently. On the contrary, language that exaggerates the importance of findings may fuel skepticism and alienate the reader. Perhaps journals should encourage more modest claims on the part of the authors and encourage a return to objectivity. To end at the beginning; "The numbers and not their interpretation, must speak for themselves."

## Competing interests

The authors declare that they have no competing interests.

## Authors' contributions

VF contributed to the study design and performed the literature review for the assessment of the vocabulary employed and performed the analysis of the data. She prepared the first draft of the manuscript. JM contributed to the design of the study, assisted with data analysis and presentation, and wrote the final draft of the manuscript.
